# A 3D Analysis of Flight Behavior of *Anopheles gambiae sensu stricto* Malaria Mosquitoes in Response to Human Odor and Heat

**DOI:** 10.1371/journal.pone.0062995

**Published:** 2013-05-02

**Authors:** Jeroen Spitzen, Cornelis W. Spoor, Fabrizio Grieco, Cajo ter Braak, Jacob Beeuwkes, Sjaak P. van Brugge, Sander Kranenbarg, Lucas P. J. J. Noldus, Johan L. van Leeuwen, Willem Takken

**Affiliations:** 1 Laboratory of Entomology, Wageningen University, Wageningen, The Netherlands; 2 Experimental Zoology Group, Wageningen University, Wageningen, The Netherlands; 3 Noldus Information Technology BV, Wageningen, The Netherlands; 4 Biometris, Department of Mathematical and Statistical Methods, Wageningen UR, Wageningen, The Netherlands; 5 Facility Services, Wageningen University, Wageningen, The Netherlands; Johns Hopkins University, Bloomberg School of Public Health, United States of America

## Abstract

Female mosquitoes use odor and heat as cues to navigate to a suitable landing site on their blood host. The way these cues affect flight behavior and modulate anemotactic responses, however, is poorly understood. We studied in-flight behavioral responses of females of the nocturnal malaria mosquito *Anopheles gambiae sensu stricto* to human odor and heat. Flight-path characteristics in a wind tunnel (flow 20 cm/s) were quantified in three dimensions. With wind as the only stimulus (control), short and close to straight upwind flights were recorded. With heat alone, flights were similarly short and direct. The presence of human odor, in contrast, caused prolonged and highly convoluted flight patterns. The combination of odor+heat resulted in longer flights with more landings on the source than to either cue alone. Flight speed was greatest (mean groundspeed 27.2 cm/s) for odor+heat. Odor alone resulted in decreased flight speed when mosquitoes arrived within 30 cm of the source whereas mosquitoes exposed to odor+heat maintained a high flight speed while flying in the odor plume, until they arrived within 15 cm of the source. Human odor evoked an increase in crosswind flights with an additive effect of heat at close range (<15 cm) to the source. This was found for both horizontal and vertical flight components. However, mosquitoes nevertheless made upwind progress when flying in the odor+heat generated plume, suggesting that mosquitoes scan their environment intensively while they progress upwind towards their host. These observations may help to improve the efficacy of trapping systems for malaria mosquitoes by (1) optimizing the site of odor release relative to trap entry and (2) adding a heat source which enhances a landing response.

## Introduction

Insect flight is mediated by a wide range of sensory cues [1], [2]. During upwind flight, these may, e.g., induce a visually-guided, odor-modulated anemotaxis, which aids the insect in reaching the source of a stimulus. For the nocturnal African malaria mosquito, *Anopheles gambiae* Giles *sensu stricto* (hereafter referred to as *An. gambiae*), chemical cues are thought to be most important, directing the insect upwind from a distance to a blood host [3]–[5]. Chemical stimuli from the host may inform the mosquito not only about the location of the host, but also of its quality [6]. Mosquitoes can be attracted by (host-) odors alone [5], [7], [8], but surrounding visual and mechanical cues are important for determining flight direction in insects, even for nocturnal species under low light conditions [9].

For many insects, flight is affected by pheromones, released from an approximate point source [10], [11]. The insect reaches the source by navigating upwind while making reiterative contact with packets of odor in a relatively narrow odor plume [12]. The physical properties of the plume depend of course strongly on the wind conditions. Insects may also respond to blends of kairomones (which originate from one or more other species than that of the receiver). Examples are hematophagous mosquitoes, sandflies, biting midges and tsetse flies [4], [13]–[17], and herbivorous insects such as common fruit flies, aphids and Colorado potato beetles, which navigate in a broad plume of odor-laden air, facilitating upwind orientation [18]–[20]. Kairomones often originate from a wider source than pheromones, resulting in a much broader plume even close to the source. These differences may also affect fine-scale characteristics of the odorants in the plume, and affect the insect behavioral response.

Odor composition influences the efficacy of host-seeking behavior during upwind odor-modulated flight in the mosquitoes *Culex quinquefasciatus* Say, *Cx. tarsalis* Coquillet and *Aedes aegypti* L. [21], [22]. For example, odor composition affects the frequency and extent of crosswind flight behaviors in these species at certain distances from the source. For *An. gambiae*, however, it is not clear how olfactory cues affect flight characteristics and therefore host-finding efficacy [2], [23]. In the absence of olfactory cues, it was found that two *Anopheles* species tended to fly upwind in an approximately straight path [24].

The sensory cue heat elicits probing behavior in *Aedes* mosquitoes, but its precise role in host-seeking remains unclear [25]. Heat also affects the mosquito at close proximity to the host [26], but whether the addition of heat enhances host finding remains unresolved because its effect on mosquito behavior has often been studied in combination with moisture and odor [27]. For example *Cx. quinquefasciatus* flew less directly upwind when exposed to human odor plus heat compared to its flight pattern in clean air [7]. Radiated heat from a source can only be detected at close range [28], [29], because the effect of the radiated heat on air temperature declines rapidly with distance. In contrast, odors can be detected from a much greater distance from the host [23], [30], [31]. The combined effect of odor and heat on mosquito behavior has rarely been studied. Details of in-flight characteristics when the insects are exposed to both stimuli simultaneously may elucidate behaviors that explain how both stimuli interact in guiding mosquitoes to their blood source.

Advances in automated tracking tools have made it possible to investigate the behavior of nocturnal insects such as *An. gambiae* in great detail [32]. The aim of the current study was to elucidate characteristics of the host-seeking process of the malaria mosquito *An. gambiae* that can be exploited to develop species-specific trapping methods [33]. Flight characteristics of mosquitoes exposed to host stimuli were studied by 3D tracking individual insects while they navigated through a plume of host-emitted cues in a wind tunnel under nocturnal conditions. We present a quantitative analysis of the flight response of *An. gambiae* in the presence of a source of either human odor or heat, or a combination of the two stimuli. We expected that flight speed and angles of approach would vary with the distances from the source (either odor, heat or a combination) and the location of the insect with respect to the (approximate) extent of the odor plume. The detailed flight analysis provided insight into key characteristics that shape the behavioral response of a nocturnal mosquito to host-specific cues.

## Materials and Methods

### Mosquitoes

The *An. gambiae* colony at Wageningen University, the Netherlands, originated from Suakoko, Liberia in 1987 and has been reared on human blood since 1988. Ethical approval for blood feeding was not requested as the authors did not consider this to be subject to the Dutch Act of Medical Research involving Human Subjects (WMO). Blood feeding was considered to cause a medium risk of allergic reaction and provision was in place that individuals were excluded if they reacted strongly to bites. In our anopheline mosquito cultures, no experimental infections took place and mosquitoes were free of any parasite. The colony was maintained at 27±1°C, 70±5% R.H., and a light cycle of LD12∶12 h. Adults were fed with a 6% glucose solution *ad libitum* and offered a blood meal twice a week for 10 min. Females laid eggs on wet filter paper and these were transferred to water trays before hatching. Larvae were fed Tetramin® (Tetrawerke, Melle, Germany) fish food daily.

### Wind Tunnel and Air Treatment System

**Figure 1:**
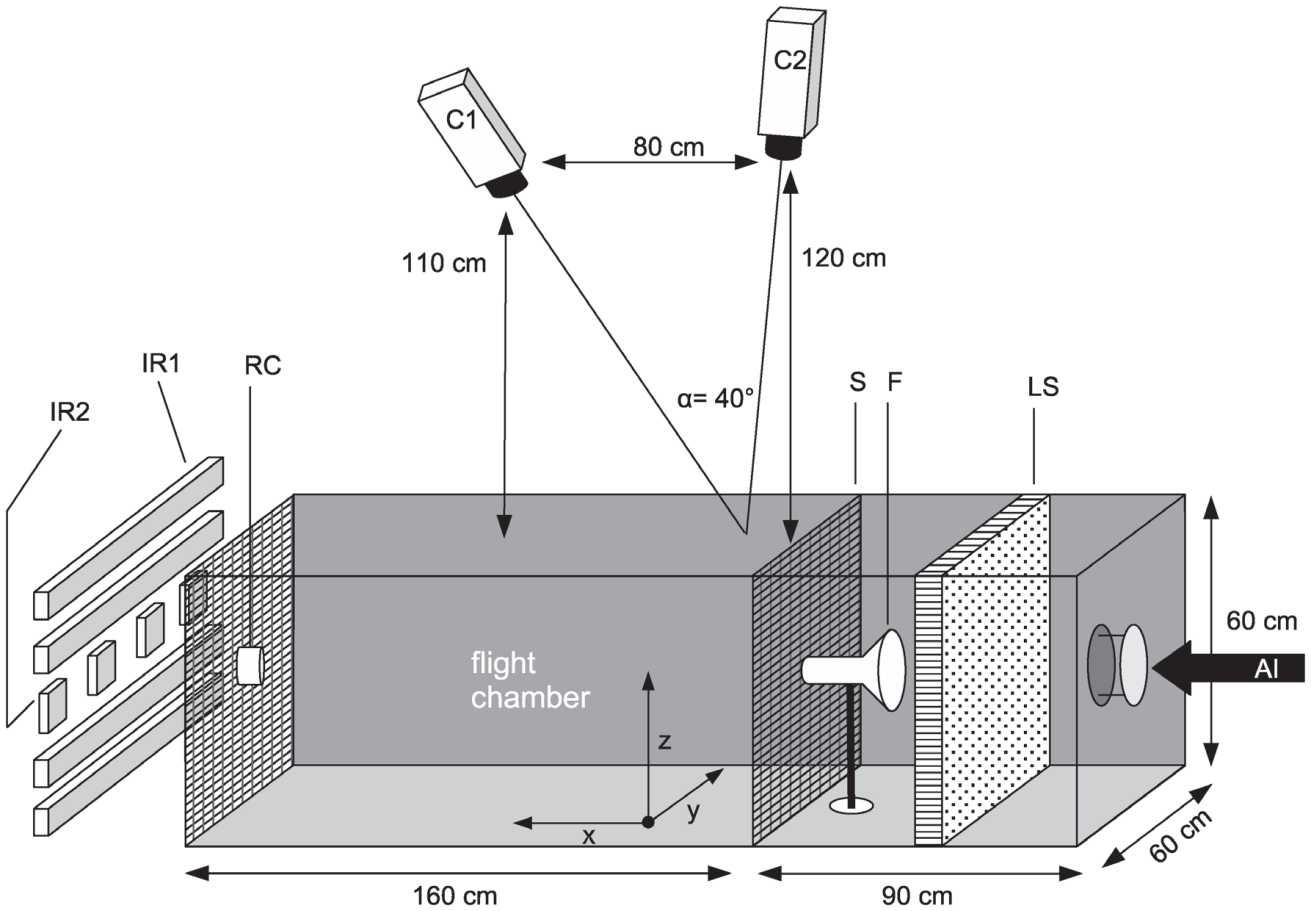
Schematic diagram of wind tunnel. Air inlet (AI), lamination screen (LS), glass funnel containing heat element (F), mesh screen (S), release cup (RC), cameras (C1,2), IR lights type 1 (IR1), IR lights type 2 (IR2). The IR2 lights were operated by setting the accompanying adaptors at 9 Volts.

**Figure 2:**
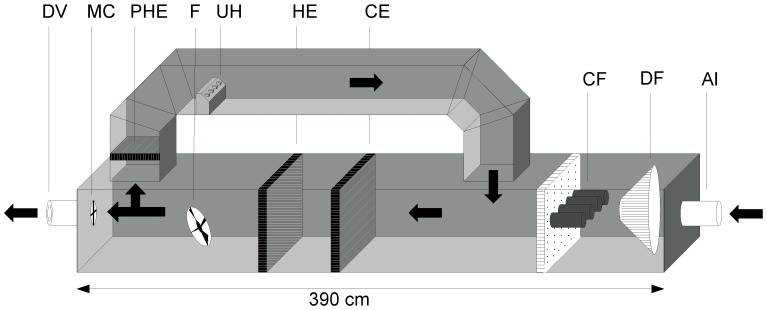
Schematic diagram of air treatment system. Air inlet (AI), fine dust filter (DF), charcoal filter cartridges (CF), cooling element (CE), heating element (HE), ultrasonic humidifier (UH), fan (F), pre-heating element (PHE), measuring cross (MC), diaphragm valve (DV).

All experiments were conducted in a wind tunnel (160 cm long and 60×60 cm across), supplied with air by a computer-controlled air treatment system (Figure 1 and Figure 2) at 27±1°C, 70±3% relative humidity and a wind speed of 20.0±1.0 cm/s. A black epoxy metal mesh screen covered both ends of the wind tunnel. A glass funnel (diameter of straight tube, 4.3 cm; diameter at open top, 10.5 cm) was used to create an odor plume that was positioned in the center of the screen at the upwind end of the tunnel. A cylindrical brass heating element was placed horizontally inside the funnel. Mosquitoes were released individually from a device placed in the center of the downwind screen. For details see Text S1.

### 3D Flight Recording

Infrared (IR) light units were placed at the downwind end of the wind tunnel, facing upwind. The reflection of IR light from the mosquitoes’ wings was filmed with two Cohu 4722–2000/0000 monochrome CCD video cameras (Cohu, San Diego, CA, USA) with Fuji non-tv f1.4 9 mm lenses, synchronized within 0.01 s. MPEG-4 videos (PAL video: 704×576 pixels interlaced at 25 frames/s) were obtained for offline tracking. A software package, “Track3D” (Noldus Information Technology, Wageningen, The Netherlands) was developed as an add-on tool to EthoVision 3.1 to produce 3D track data for the position of a mosquito every 0.04 s from the 2D coordinates obtained from the two cameras. The air velocity in the wind tunnel was set at 20.0 cm/s in the positive *x* direction. The flight velocity was calculated by time differentiation of a spline function through a sequence of target positions (block), see Table S1. A flight angle of 0 degrees is defined as exact upwind flight. Speed is defined as the magnitude of the velocity vector in the 3D space and presented in a coordinate system that is fixed to the boundaries of the flight arena. Angular change is the change in the angle in space formed by successive data points. The mean angular velocity over the sample time is the angular change of the velocity vector times the sample frequency. We identified sequences of flight tracks that occurred a) within the defined space of the odor plume, 2) within a transition zone of 2.5 cm directly outside the plume, or 3) outside the transition zone (i.e., in notionally ‘clean’ air). The odor plume was approximated as a cone in space. Its dimensions were estimated by releasing smoke produced by a Safex® fog generator, F2010^plus^ (Safex-Chemie Gmbh, Schenefeld, Germany) using perfume-free fog fluid. The estimated extent of the odor plume was defined by the apex, a point on the axis, and the cone angle of the funnel described above. Plume dimensions were estimated for treatments both with and without a heat source, while correcting for the effects of convection currents indicated by visualization experiments with smoke. The cone description is an approximation, as its boundaries are in fact variable. Details of computational methods used to produce 3D track data are provided in the Text S2.

### Experimental Procedure

Individual 5–7 d-old female mosquitoes, which had not received a blood meal, were transferred to release containers 14–16 h before testing. A water-soaked cotton wick was placed in the container to prevent desiccation of the mosquito. Experiments were conducted during the last 4 hours of the scotophase. Mosquitoes that were not within the field of view of both cameras within 3 min after release were recorded as ‘no response’. Recordings were stopped 10 min. after the first entry of the mosquito within camera view or earlier if the mosquito landed on a wall or the upwind screen for ≥3 s. Mosquito response was categorized as either ‘no response’, ‘landing on upwind screen’, landing at ‘the source’ or landing ‘elsewhere’ in the arena. A new mosquito was used for each bioassay. Surgical gloves were worn to avoid contamination of the experimental equipment.

### Stimuli

Mosquito flights were recorded in the presence of combinations of four stimuli; ‘No odor, no heat’ was tested as the control, with a clean nylon sock placed in the glass funnel and the heat element switched off. The same set-up was used for the treatment ‘heat’, but with the heat element set at 34°C to mimic human skin temperature. Thirdly, ‘odor’ was tested by using a nylon sock (worn for 24 h by JS), containing foot odors [34]. The combination of odor and heat was designated ‘odor+heat’. Female *An. gambiae* are attracted to human volatiles present on a nylon sock [34], [35]. To minimize variation in odor over days, the sock was re-used and stored in a freezer between testing days. Each day, treatments were changed after testing five mosquitoes. The testing order was randomized between and within days to rule out effects of testing sequence.

### Data Analysis

An area of 60×60×60 cm at the upwind end of the wind tunnel was in view of both cameras. Data points acquired ≤2 cm from the walls, including the upwind screen, were discarded for analysis because these boundaries may have affected flight parameters. Track duration was defined as the total time a mosquito was within the field of view of both cameras and within the area of interest, thus excluding flight within 2 cm of boundaries. A Chi-square test was used to examine differences in response between treatments. A two-tailed t*-*test with unequal variance served to compare the proportion of time spent in different ‘zones’ of the flight arena: inside the plume, the transition zone or outside the plume for mosquitoes that landed in the proximity of the odor+heat source on the upwind screen (5.0 cm diameter) with those landing elsewhere on the upwind screen.

Differences in flight parameters between treatments or between different ‘sections’ of 15 cm increments along the x axis from the upwind screen within a treatment were tested with a GLM, Tukey (T) or Games Howell test depending on the equality of variances (SPSS 19.0.1 for Windows). Non-normal data such as the proportion of time in a certain zone or angular velocity magnitude were tested with a Mann-Whitney U (two groups) and Kruskal-Wallis test (four groups). Mosquitoes that did not land on the upwind screen were excluded from the analysis that tested differences between the sections. Differences were analyzed only for flights containing a minimum of 20 recorded positions of the mosquito in the two image planes. This was reduced to six positions for the analysis between the 15 cm sections, where fast (up to 75 cm/s straight upwind) flying mosquitoes would otherwise be excluded from the results. An independent sample t-test was used to test for differences between flight speeds in- and outside the plume. The square root of this variable was taken to normalize the data.

Comparisons between flying in- and outside the plume (with exclusion of the transition zone) were only done for the treatment odor+heat, as for the other treatments there were insufficient data for within-plume flights. The change in flight speed was analyzed in more detail with a paired sample t-test by comparing the average flight speed of 10 frames before entering the transition zone around the plume with the following 10 frames after the transition zone. The distance to the upwind screen when entering a plume was compared with the distance at exiting (and vice versa) and tested for significant differences using a Wilcoxon test.

Distribution of flight directions was presented in rose diagrams and analyzed for the *xy* and *xz* plane over 3° bins and tested for significant differences between treatments using GLMs. Rose diagrams were computed by using the weighted mean absolute cosine and absolute sine across bins, with the sum of velocities of each mosquito in each bin as weights. Parallel to the *xy* plane this absolute sine measures the frequency of crosswind flight and the absolute cosine the frequency of up/down wind flight. The ratio of absolute sine and cosine (the absolute tangent) expresses the degree of crosswind maneuvers. A high absolute tangent indicates strong crosswind movement and a low one dominant up/down- wind fly movement.

The tangent was analyzed in three ways. As the experiment had a 2×2 factorial design of factor “odor” and factor “heat”, both with two levels (no, yes), the first analysis was a GLM of an ANOVA type and examined the main effect of odor (averaging over heat levels) and of heat (averaging over odor levels) and the interaction between odor and heat which measures whether the odor effect depends on the heat level. As the interaction was non-significant, the combined effect of heat and odor was the sum of the main effect of odor and that of heat. Secondly, paired t-tests were performed to compare the absolute tangent of flight paths in plume vs. outside plume for A) all mosquitoes exposed to odor+heat and B) the mosquitoes that landed on the odor+heat source. Third, a linear mixed model analysis was performed to study whether the mosquitoes that landed in the proximity of the source showed a different flight path than mosquitoes that did not. This was done irrespective of the mosquito being in or outside the plume, while correcting for a presumed effect of being in or outside plume.

## Results

**Figure 3:**
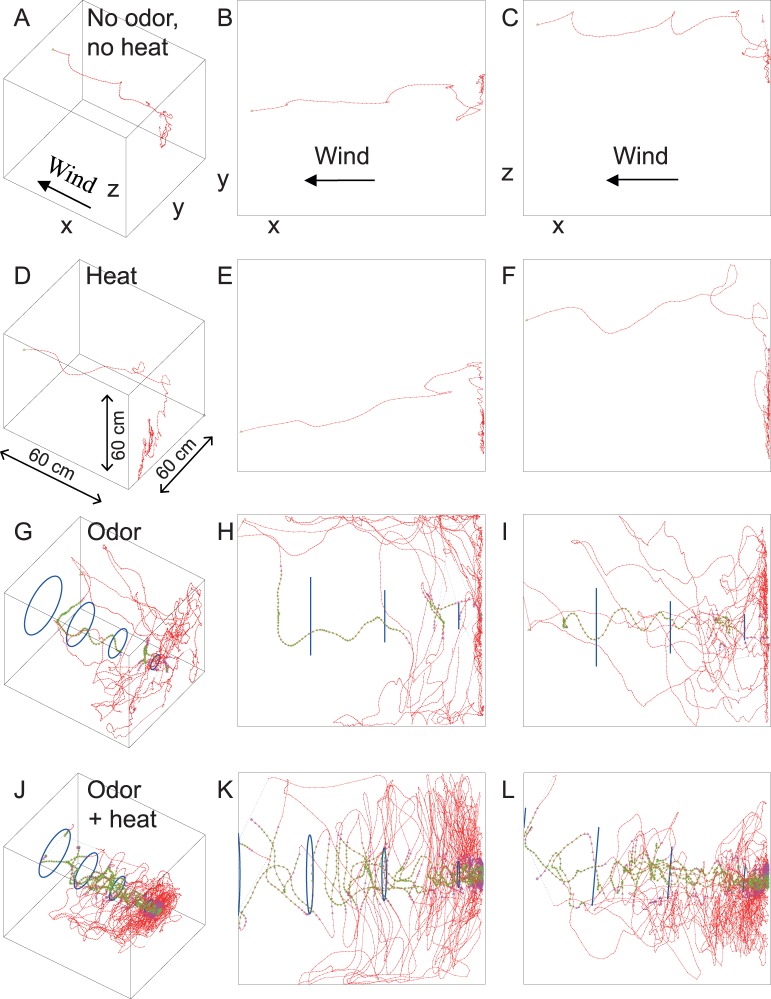
Examples of flight tracks of *Anopheles gambiae s.s.* for each treatment viewed from different angles. Each treatment represents a single female. A–C clean acclimatized air only (control, 9 s), D–F heat (21 s), G–I human odor (112 s) and J–L human odor+heat (231 s). Red dots indicate samples outside the cone and transition zone, magenta triangles are used for samples within the transition zone and green stars indicate that the insect is tracked within the defined odor plume. Mosquitoes that landed near the center of the upwind screen, within a circle with a diameter of 5 cm, were recorded as landing on the source.

Flight tracks were reconstructed into 3D images as shown in Figure 3. As a considerable proportion of mosquitoes exhibited displacements both in *xy* and *xz* planes, a 3D analysis was required to estimate behavioral parameters with sufficient accuracy. The trajectories showed that without a host cue, many mosquitoes flew upwind, along the x-axis, with relatively little deviation in the y- and z-axis, followed by landing on the upwind screen (Figure 3A–C). With heat alone, flights were similarly short and direct (Figure 3D–F). The presence of human odor, by contrast, caused longer and highly convoluted flight patterns (Figure 3G–L).

The reconstructed plume was cone shaped with an estimated cone angle of 20°. With heat (34°C), the angle of the plume relative to the floor was estimated to be 13°. To account for variations in plume eddies, a transition zone of 2.5 cm from plume to outside plume was defined and data points of mosquitoes flying in this zone were excluded from the analysis concerning ‘in plume’ or ‘outside plume’ behavior. Temperature readings showed that the heat element heat affected air temperature up to 20 cm downwind from the source (Figure S1).

**Figure 4:**
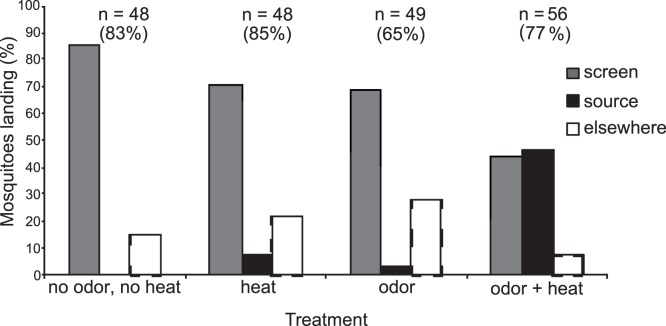
Mosquito responses with four different treatments. The percentage of responding mosquitoes landing on the upwind screen, source, or elsewhere in the arena per treatment. n = Number of mosquitoes tested. Percentage (%) = percentage of mosquitoes leaving the release site within 3 min.

Of the 201 mosquitoes tested, 156 (78%) responded by flying upwind and 145 of the tracks were recorded and used for analyses. An upwind flight response varied from 65% to 85% (Figure 4) and was not significantly different between the treatments (χ^2^, *P* = 0.076). Human odor alone caused the lowest flight response (65%) within 3 min., and up to 67% of all responding mosquitoes landed on the upwind screen. Odor+heat elicited landing within 5 cm of the center of the source for 46.5% of the responding mosquitoes, which is more than the other treatments, in which 0–7% of the mosquitoes landed at the source (χ^2^, *P*<0.05, Figure 4). The percentage of landings on the odor+heat source was similar during the first five (41%) and the last five testing days (48%), indicating that the storage and re-use of the worn sock did not affect responses over time (χ^2^, *P* = 0.458). Mosquitoes exposed to odor+heat that landed on the source (n = 20) spent significantly more time flying than mosquitoes that landed elsewhere on the upwind screen (n = 99; 99±23 s vs. 17±3.2 s, Mann Whitney U test, *P*<0.001).

Flight tracks were longer if mosquitoes were exposed to human odor+heat compared to heat alone or the control (Table 1, GLM, *P*<0.05). The percentage of time spent inside the plume was greater for mosquitoes that landed on the odor+heat source than those exposed to the same stimuli, but landing elsewhere on the upwind screen (Table 2, two-tailed *t*-test, *P*<0.01).

**Table 1:** Mean (± s.e.m.) track duration, mean up- and downwind flight velocity components and magnitude of angular velocity of mosquitoes that flew upwind in the defined arena exposed to four different treatments.

Treatment	track duration		speed upwind		speed downwind		ang. velocity for upw. flight		
	n	(s)	n	(cm/s)	n	(cm/s)	n		(degrees/s)
**no odor, no heat**	33	15.2±9.8 a^2^	30	22.7±1.2 a	18	20.4±1.7a^1^	30	465.9±31.3 a^2,3^	
**Heat**	41	18.8±8.8 a^2^	39	24.7±1.0 a	25	25.4±1.5 ab^1^	39	556.9±27.4 a	
**Odor**	32	32.7±9.9 ab^2^	30	24.3±1.2 a	24	25.9±1.5 b^1^	30	508.1±18.5 a	
**odor+heat**	39	67.3±8.9 b^2^	36	25.9±1.1 a	27	27.2±1.4 b^1^	36	553.0±27.8 a	

Flight speed is defined as the mean speed relative to the boundaries of the wind tunnel over all time intervals with an upwind and downwind velocity component. Wind speed was 20 cm/s. Different letters within a column indicate significant differences between treatments (GLM, *P*<0.05). n = Number of included flights.

1 Equal variances Tukey (T),

2 Unequal variances Games Howell.

3 *P = *0.057.

**Table 2:** Mean (± s.e.m.) percentage of time mosquitoes spent inside the plume, in the transition zone or outside the plume boundaries as defined for the treatment odor+heat.

mosquitoes landing:	n	mean (%) of time spent flying in a zone of the odor plume		
		inside plume	transition zone	outside plume
**on the source**	16	30.5 (±3.5) a	8.1 (±0.7) a	61.4 (±3.9) a
**on upwind screen, away from the source**	19	7.3 (±2.9) b	3.8 (±1.0) b	88.9 (±3.8) b

n = Number of included flights. Different letters in the same column express significant differences in time spent in the corresponding section for mosquitoes that landed eventually on the source compared to mosquitoes that landed elsewhere on the upwind screen (two tailed *t*- test with unequal variance (*P*<0.01)).

### Flight Direction

**Figure 5:**
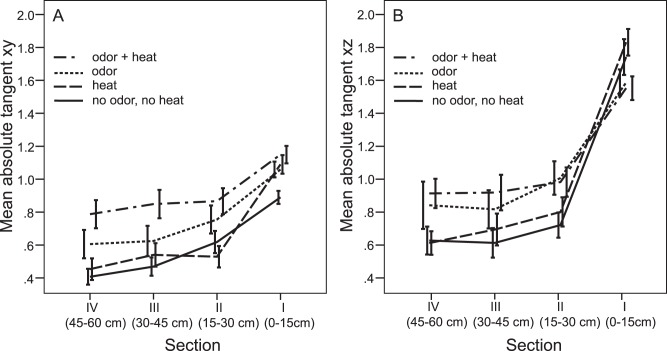
The degree of crosswind flight plotted for the horizontal (*xy)* and the vertical (*xz*) plane. Mean (± s.e.m.) absolute tangent of mosquito flight paths at four different sections from the upwind screen for each treatment including the control (no odor, no heat).

Vertical and horizontal crosswind flights were common for all treatments when mosquitoes flew close to the upwind screen. At distances >15 cm from the upwind screen, linear up- and downwind movements dominated for all treatments other than treatment odor+heat (Figure S2 and Figure S3). For the latter, crosswind flights dominated, especially 15–30 cm from the upwind screen, and further away from the source, these mosquitoes more often expressed downwind-directed flights than those exposed to one of the other three treatments. Human odor contributed most to the observed crosswind movements (Table S2) and this was especially apparent for horizontal (*xy*) movements and significant (*P*<0.001) for all sections. Close to the source (<15 cm), the change in crosswind behavior was also influenced by heat. For vertical (*xz*) flights, this change was, within each ‘15 cm’ section, significantly affected by odor but not by heat. There was no interaction between the treatments odor and heat (*P>*0.05). Because both cues caused a positive effect on the absolute tangent, odor+heat showed, of all treatments, the largest increase in crosswind maneuvers compared to the control. This is summarized in Figure 5 where the mean (± s.e.m.) tangent is presented per section for the horizontal and vertical plane.

The mean magnitude of angular velocity for upwind flight was not different between any of the treatments for upwind flight (GLM, *P* = 0.057, Table 1) and for downwind flight (GLM, *P = *0.480). When all treatments are pooled, mosquitoes flew downwind with a greater mean magnitude of angular velocity compared to upwind flights (653.4±19.7 (n = 94) vs. 524.8±14.9 (n = 135) deg/s, respectively; Mann-Whitney U test, *P*<0.001). Mosquitoes had the highest mean angular velocity magnitude when at <15 cm from the upwind screen. (Kruskal-Wallis, *P*<0.001).

During upwind flight, the exposure to odor+heat led to more crosswind movements outside than inside the plume (Table 3A). This was most apparent at <30 cm from the screen but not significantly different between the 15 cm sections. The same trend was visible for mosquitoes that landed on the source (Table 3B). Mosquitoes landing on the odor+heat source expressed overall more crosswind behavior than those landing elsewhere on the screen irrespective of flying in or out the plume (Table 3C). This was observed for all spatial sections and for both horizontal and vertical flight components except for the *xz* plane in section I (<15 cm).

**Table 3:** Statistics for different flight sections where positive estimates (± s.e.m) represent a higher tangent outside the plume compared to inside (A,B) and a higher tangent when landing on the source compared to landing elsewhere (C).

paired t-test unweighted analyses	A) in plume vs. outside plume, all mosquitoes					B) in plume vs. outside plume, mosquitoes landed on source					Linear mixed model	C) mosquitoes landed on source vs. mosquitoes that landed elsewhere, irrespective of plume			
section (cm)		xy		xz		Xy			xz		xy			xz	
	df	Est.	s.e.m.	Est.	s.e.m.	df	Est.	s.e.m.	Est.	s.e.m.	Est.		s.e.m.	Est.	s.e.m.
**I–IV 0–60**	23	0.131**	0.045	0.171**	0.051	15	0.095	0.055	0.118.	0.06	0.396***		0.088	0.299**	0.114
**I <15**	21	0.111	0.112	0.164	0.116	14	0.173	0.123	0.132	0.126	0.220*		0.097	0.058	0.145
**II 15–30**	18	0.245	0.163	0.235	0.163	14	0.143	0.107	0.157	0.157	0.329*		0.164	0.394*	0.166
**III 30–45**	19	−0.025	0.21	−0.022	0.178	15	−0.088	0.253	−0.044	0.202	0.611***		0.155	0.775***	0.177
**IV 45–60**	19	0.145	0.12	0.033	0.134	13	0.029	0.136	−0.109	0.133	0.650***		0.145	0.600***	0.143

Paired t-test results for the treatment odor+heat with the difference in tangent while in the plume vs. out the plume (A) and the difference between in plume vs. out plume, only for mosquitoes that landed on the source (B). The difference in tangent for the mosquitoes that landed on the source compared to mosquitoes that landed elsewhere on the upwind screen, irrespective of the plume, using a linear mixed model (C). df Represents the degrees of freedom (n−1). Signif. codes: 0 ‘***’ 0.001 ‘**’ 0.01 ‘*’ 0. 05 ‘.’ 0.1.

**Figure 6:**
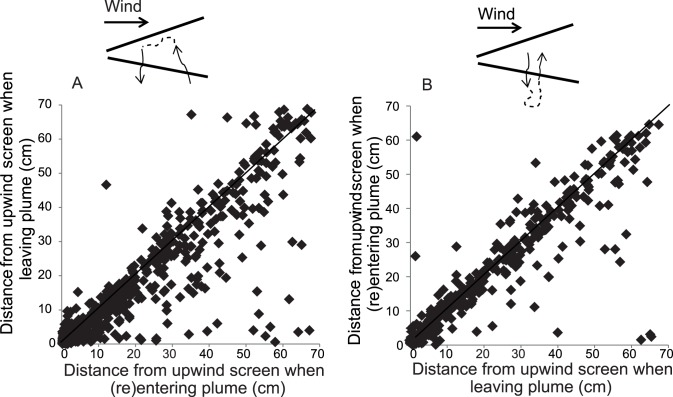
Displacement of ‘odor+heat’ treatment mosquitoes along the *x* axis of the wind tunnel in relation to entering and leaving the plume. A - Distance from the upwind screen at the moment of entering the plume (*x* axis) and the moment of leaving (*y* axis). B - Distance from the upwind screen at the moment of flying outside the plume and (re-) contacting the plume. The figure represents all individuals (n = 16) that landed near the odor+heat source. The absolute number of occurrences per mosquito is given in table S3. The solid line represents a theoretical situation where net up- or downwind displacement between the moments of entering and leaving, or vice versa is equal.

By comparing their *x*-positions from the moment mosquitoes entered until they exited the plume it was found that the insects made upwind progress when flying in the plume (Figure 6A, Wilcoxon, Z_−6.921_, P<0.001). This was observed for mosquitoes that reached the source and also for those that landed elsewhere on the upwind screen (data not shown). No directional bias was observed after comparing the *x*-position when outside the plume until (re-)contacting the plume (Figure 6B, Wilcoxon, Z_−0.669_, P = 0.504), which confirms our data of increased crosswind behavior for flights outside the plume (e.g. Table 3). The mosquitoes still flew forward with respect to the air when outside the plume because we have considered ground speeds. We refer to Table S3, for the mean upwind progress per mosquito.

### Flight Speed

**Figure 7:**
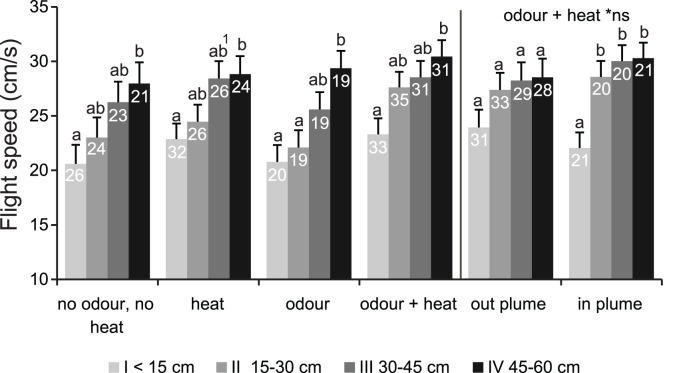
Mean (± s.e.m.) flight speed (cm/s) over all time intervals with an upwind velocity component in different sections from the upwind screen. Flight speed is relative to the boundaries of the wind tunnel. Wind speed was 20 cm/s. Different letters indicate significant differences between sections (GLM, Equal variances Tukey (T), *P*<0.05, ^1^ *P* = 0.051). Numbers inside bars represent the number of included flights. The flight speed for the treatment odor+heat is also presented while flying outside the plume and inside the plume, respectively. * Differences between out plume and in plume speed, within a distance section, were not significant (ns, independent sample t-tests, *P*>0.05).

Mosquitoes, pooled over all treatments, flew upwind with a mean speed relative to the ground of 24.5±0.6 cm/s (n = 135), and downwind at 25.1±0.8 cm/s (n = 94). Flights of mosquitoes exposed to odor+heat (27.2±1.2 cm/s, n = 38) were significantly faster than those of control mosquitoes (22.5±1.29 cm/s, n = 30, GLM, *P*<0.05). The difference in flight speed between the treatments is mainly caused by downwind flights, with a lower flight speed of control mosquitoes than those exposed to odor+heat (Table 1, *P*<0.05). Within the different treatments, upwind flight speed was reduced when reaching the upwind screen (Figure 7). This was significant between the sections 0–15 cm and 45–60 cm. Odor alone, however, also caused reduced flight speeds between the section 15–30 cm and 45–60 cm. At 15–30 cm, flight speed was significantly higher for odor+heat (27.6±14 cm/s) than for the odor treatment (22.1±1.6 cm/s, GLM, *P*<0.05). For odor+heat, which elicited 46.5% of the mosquitoes to land near the source, mosquitoes maintained their relatively high flight speeds up to 15 cm from the upwind screen. A strong decrease in flight speed at <15 cm was observed only for in-plume mosquitoes (*P*<0.01, Figure 7). At this distance the mean upwind flight speed was 23% lower than at 15–30 cm from the screen and lower in the plume than outside it. For the three sections further downwind, the differences in flight speed were not significant (independent sample t-tests, P>0.05). Insufficient replicates were available to examine flight speed per section within the plume for the other treatments. A considerable change in flight speed (either negative or positive) occurred upon entering or leaving the plume (paired sample t-test, t (141) = 0.41, *P* = 0.67; t (169) = −0.53, *P* = 0.60, respectively). Mean flight speeds upon entering were overall 4.5 cm/s higher than those at the moment of leaving the plume (GLM, *P*<0.001, visualized in Figure S4).

## Discussion

The analysis of upwind odor-modulated anemotaxis of the nocturnal mosquito *An. gambiae* revealed significant differences in flight behavior between the treatments odor, heat and odor+heat, especially at <30 cm from the source. Without additional cues, mosquitoes took off for upwind flight as described by Cardé and Gibson and references therein [23]. Odor+heat caused a high landing response on the source. This was preceded by a longer, faster and more tortuous flight than with odor or heat treatments as single cues, suggesting that with the combination a more effective behavior is induced, enabling the insect to locate the host more precisely. Human odor contributed most to the observed crosswind behavior. Close to the source, heat also induced an increase in crosswind movements and flight speed was reduced.

### Flight Direction

Without odor, many insects fly crosswind or downwind [36]. When odor is lost during upwind flight, insects generally exhibit casting behavior, which is described as back and forth movements across the wind without making much progress upwind [2], [23].Upon restoration of contact with odor, they continue flying upwind [37]. Tsetse flies, however, respond to odor plumes by turning sharply upwind when flying into a plume [13], [38]. The mosquitoes *Cx. tarsalis* and *Cx. quinquefasciatus*, when approaching an odor-baited trap, flew straight upwind until they overshot the odor source, at which point they engaged in more tortuous flight [39]. When these mosquitoes approached a source of CO_2_, heat and moisture, the tortuosity of the flight path increased and speed reduced within 30 cm of the source. Increased tortuous flights in response to host stimuli are also described for *Ae. aegypti* [40] and *Cx. quinquefasciatus* [7]. This is similar to our observations with *An. gambiae* approaching odor+heat and may be a general behavioral pattern when mosquitoes arrive in proximity of a host. The increased crosswind behavior suggests that upwind navigating host-seeking mosquitoes scan the arena before landing on the host. Mosquitoes that did not land on the source spent more time outside the plume (Table 2). Some of them spent little to no time in the plume and continued flying upwind, which may explain the results shown in Table 3C. This also suggests that the observed increase in crosswind maneuvers while outside the plume (Table 3AB) was triggered by initial encounters with the plume. These findings validate a recent simulation study of mosquito flight behavior in response to odor cues [41]. The authors suggest that the crosswind strategy led to the largest percentage of host finding, but that such flights took substantially longer. Our results are in agreement with these conclusions, as crosswind flights increased significantly in odor and led to longer flight trajectories.

### Flight Speed

Dekker et al. [22] reported higher velocities for *Ae. aegypti* when exposed to skin odor or a human hand than to clean air. They mention that flight speed remained close to constant, irrespective of flying in or outside the plume. We observed a higher mean flight speed for the odor+heat treatment than for clean air. Flight speed decreased significantly in the vicinity of the odor+heat source (Figure 7). Lacey and Cardé [7] found that flight speed of *Cx. quinquefasciatus* was lower when exposed to heated foot odor compared to heat alone, but here flight speed varied within all treatments at different distances from the source. Our observed difference in average flight speed just after entering a plume compared to after leaving it suggests that *An. gambiae* mosquitoes reduce ground speed after entering the odor plume and fly more upwind. Flying more upwind reduces ground speed even if the mosquito maintains the same flight speed with respect to the air. With the current approach it takes more than 0.9 seconds before we can measure this change in flight speed (20 frames = 0.8 s+ca. 0.1 s in the transition zone). The proportion of time spent in the plume was significantly higher for mosquitoes that landed on the odor+heat source than for those that landed elsewhere on the upwind screen. Moreover, when the odor+heat source was approached, flight speed decreased and the angular velocity increased, suggesting that the insect made many turns per second to assess the exact location or quality of the odor plume, which is considerably narrower at this point than further downwind (Figure 3). Both behavioral changes are most likely an adaptation to prepare for a landing on or near the host [28]. These results demonstrate that contact with odor causes a significant shift in behavior, allowing the insect to more accurately assess the source of the host stimuli.

The behavior of mosquitoes close to the upwind screen suggests that the screen acts as a physical barrier, which is sensed from a (short) distance without the need for making contact with it. We assume that this may be caused by a change in wind structure due to increased air turbulence around the mesh screen or visual cues from this screen.

The response to heat, either alone or combined with odors, has previously been studied with *Aedes* mosquitoes [29] and with *An. gambiae* [27], [28]. In most studies, enhanced attractiveness was observed. However, the observations did not reveal whether heat activates odor components and therefore indirectly attracts more mosquitoes or that heat itself elicits a landing or trap entry response at close range. This last hypothesis is supported by Healy and Copland [31], who reported increased landing responses when combining heat+volatizing acids such as 2-oxopentanoic acid. Heat is likely to affect the ratio in which odorants are released from the substrate, and hence their concentration in a plume depending on molecular characteristics and this may (partly) explain our observed differences between treatments odor and odor+heat.

In *An. gambiae,* odor alone caused longer flights and increased crosswind movements compared to the control, suggesting host-seeking behavior. This effect was not observed for heat alone. This raises the question whether mosquitoes need to be in contact with host odor in order to respond to heat. *An. gambiae* have heat-sensitive molecular receptors on the antennae [42], suggesting that mosquitoes use heat sensing for host-seeking and/or recognition in addition to odor and vision. With a wind speed of 20 cm/s, the effect of body heat faded to the background over a distance of 20 cm, indicating that at greater distance from the host the principal cues for host orientation are odor and wind direction. This short range was also the distance over which *Ae. aegypti* was attracted to heat and moisture [43]. The higher flight speed observed for the treatment odor+heat at 15–30 cm, compared to mosquitoes exposed to odor alone, may be explained by an increase of odor contact due to the effects of heat on breaking up the odor filaments.

The host-seeking strategy of *An. gambiae* may be explained as consisting of highly tortuous flights, initially evoked by odor, enhancing the probability of reaching the source further upwind, whereas heat causes a significant reduction in flight speed, allowing the insect to accurately locate the source of the stimuli by a series of convoluted flights, before landing. Similar to moths, which exhibit casting behavior when losing an odor plume [11], [44], *An. gambiae* also expresses such behavior when losing odor. Casting behavior by mosquitoes, as demonstrated to occur in both *xy* and *xz* planes, is directed to lead the insects back into the plume. The dominant flight direction of mosquitoes upon plume entry is upwind (Figure 6A).

*Anopheles gambiae* females naturally orient themselves under nocturnal conditions while seeking for hosts indoors. The crosswind flights 15–30 cm from the source, by mosquitoes exposed to odor+heat (Figure S2 and Figure S3), suggest that the insects scan their environment intensively before they proceed with a landing response. This is a clear difference with diurnal mosquitoes, such as *Ae. aegypti*, which fly with less convoluted paths towards the host [40]. For other insects such as *Drosophila melanogaster* Meigen and *Manduca sexta* (Linnaeus) olfactory and visual responses are used interactively just before landing [44]–[46]. The lack of sufficient visual feedback in nocturnal endophilic insects such as *An. gambiae* may be responsible for a shift in cues which, at close range, guide them to their hosts. Lacey and Cardé [47] mention that prominent optomotor cues may indeed not affect flight orientation of the crepuscular mosquito *Cx. quinquefasciatus* while moving upwind. It remains ambiguous whether it is ortho-kinesis to heat or a different set of semiochemicals that triggers *An. gambiae* to evoke a landing response. Most likely, it is a combination of the two factors [23], [28].

The development of synthetic odor blends for mosquitoes [33]–[35], [48] in combination with 3D analysis of flight allows for detailed examination of the effect of individual olfactory components on behavior and assessment of the contribution of each component at various distances from the source. Heat strongly interacts with olfactory cues, and therefore should be considered as a potential stimulating cue when developing mosquito trapping devices. The position of the odor release point relative to the suction fan should be investigated, in order to allow for optimal dispersal of the odorants. The addition of a heat source to an odor-baited trap is likely to enhance the landing and/or trap-entry response, rendering such traps more effective. We conclude that odor and heat both affect the upwind anemotaxis of host-seeking mosquitoes. Furthermore, the adaptive flight strategy, expressed by highly convoluted flight in combination with strongly reduced flight speed in the vicinity of the source, contributes to a successful completion of this foraging process.

## Supporting Information

Figure S1 **Three series of temperature readings measured inside the flight arena.** During series 1 and 2, the thermocouples were placed in a horizontal line with the heat source, starting at the upwind screen. Series 3 was measured in positive *z* direction under an angle of 13 degrees.
(EPS)

Figure S2 **Rose-diagrams with distributions of the sum of velocities per 3° bins in the horizontal (** ***xy*** **) plane.** The sum of velocities is a measure of the total distance moved within the defined direction. The max. value is a measure of scale and represents the maximum sum of velocities plotted within the diagram. For each treatment distributions are plotted for four different sections from the upwind screen where ‘0’ represents upwind flight.
(EPS)

Figure S3 **Rose-diagrams with distributions of the sum of velocities per 3° bins in the vertical (** ***xz*** **) plane.** The sum of velocities is a measure of the total distance moved within the defined direction. The max. value is a measure of scale and represents the maximum sum of velocities plotted within the diagram. For each treatment distributions are plotted for four different sections from the upwind screen where ‘0’ represents upwind flight.
(EPS)

Figure S4 **Change in flight speed upon entering or leaving the plume.** A - The mean speed of mosquitoes exposed to odor+heat of 10 frames before entering the plume and the subsequent first 10 frames while in the plume after crossing the buffer zone of 2.5 cm (n = 17). B - Represents the mean speed of 10 frames before exiting the plume and the first 10 frames after leaving the plume (n = 19). The solid line represents a theoretical situation where mean speeds of entering/leaving are equal. The open squares show the average value of all entering/leaving occurrences per individual.
(EPS)

Table S1 **Movement parameters calculated by Track3D.**
(DOCX)

Table S2 **ANOVA for main effects and interaction of treatments on the tangent (crosswind-behavior) for different distances to the upwind screen.**
(DOCX)

Table S3 **The mean difference of ‘** ***x*** **in’ -** ***‘x*** **out’ presented for each mosquito while entering the plume and the mean difference of** ***‘x*** **out’ -** ***x*** **in’ upon exiting.**
(DOCX)

Text S1 **Air treatment system.**
(DOCX)

Text S2 **Computational methods to produce 3-D track data.**
(DOCX)
