# Supplementary material for: A 3D Analysis of Flight Behavior of Anopheles gambiae sensu stricto Malaria Mosquitoes in Response to Human Odor and Heat
Source: PLoS One. 2013 May 2;8(5):e62995. doi: 10.1371/journal.pone.0062995 (PMC3642193; doi:10.1371/journal.pone.0062995)
Supplement: Table S1 — Movement parameters calculated by Track3D. (DOCX) [file pone.0062995.s005.docx]

**
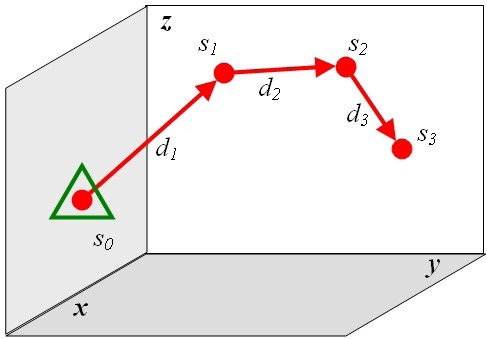
Table S1. Movement parameters calculated by Track3D.** For the complete list with information about the latest version of Track3D and associated video recording and tracking software, see  [[www.noldus.com/innovationworks/products/track3d](http://www.noldus.com/innovationworks/products/track3d).](http://www.noldus.com/track3d)

| **Name** | **Description/Formula** | **Example** |
| --- | --- | --- |
| X | The X coordinates in mm of the supposed center of the tracked animal. The axis origin (0,0,0) is the point chosen as origin during calibration |  |
| Y | The Y coordinates in mm of the supposed center of the tracked animal. The axis origin (0,0,0) is the point chosen as origin during calibration |  |
| Z | The Z coordinates in mm of the supposed center of the tracked animal. The axis origin (0,0,0) is the point chosen as origin during calibration |  |
| Plume code | Indicates where the animal's center is located relative to the supposed plume cone:  **1** — Within the cone  **2** — Within the buffer zone  **3** — Outside the cone and buffer zone |  |
| Path 3d | The distance moved from the starting point of the present block 1 up to the current sample 2. It is measured as the sum of the segments joining adjacent samples and is expressed in mm. | *Example of the path 3d parameter. Dots are sample points in the 3D space. The sample s0 is the start of the block. Path 3d equals d1 for sample s1, d1+d2 for sample s2, d1+d2+d3 for sample s3, etc.* |
| Speed | The scalar, absolute value of the velocity vector in the 3D  space. The velocity vector is calculated for each sample  *sk* with one of the three formulas, according to how many samples are present around it.  · When only one sample sk-1 occurs before sk: Velocity vk  = (pk - pk-1)/ (tk - tk-1)  · When one sample sk-1 occurs before and another sk+1  occurs after sk: Velocity vk = (pk+1 - pk-1)/ (tk+1 - tk-1)  · When more samples are available around sk (as in most cases): Velocity is the value of the derivative of a cubic spline function through all samples of that block, at sample sk.  The position vector p contains the X,Y,Z coordinates and t is the time of samples. Speed is expressed in mm/s, and can only have positive values. | 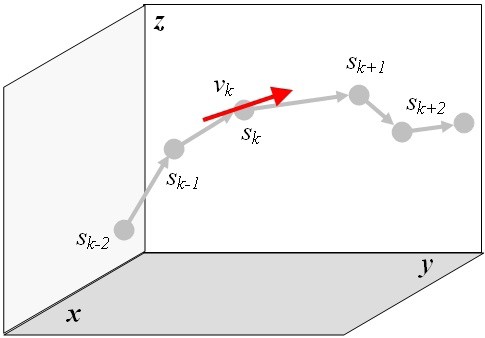  *Example of the speed parameter. Speed is the absolute value of the velocity vector vk calculated for each sample sk.* |

| **Name** | **Description/Formula** | **Example** |
| --- | --- | --- |
|  |  |  |
| Ang. ch. 3d | Stands for Angular change 3d. It is calculated as the angle in the 3D space formed by the segment joining sk and sk+1 and the prolongation of the segment joining sk and sk-1. *Angular change 3d* is expressed in degrees and can only be positive. | 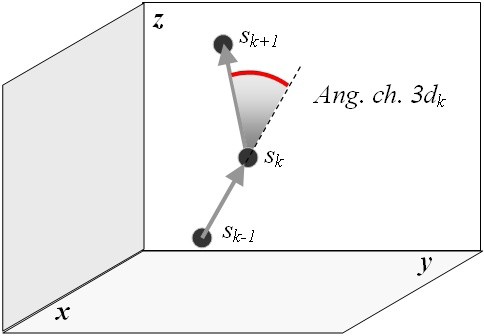  *Example of the Angular change 3d parameter. Angular change 3d is the angle in the 3D space formed by the velocity vector and the prolongation of the previous velocity vector.* |
| Ang. vel. 3d | Stands for *Angular velocity 3d*. It is the *angular change*  *3d* per time unit. It is expressed in degrees/s. |  |

*1 Uninterrupted series of valid X,Y,Z data points*

*2 Set of X,Y,Z coordinates associated with a particular time value. Corresponds to a pair of X,Y coordinates in 2- D video tracking performed by EthoVision.*
