# Supplementary material for: A 3D Analysis of Flight Behavior of Anopheles gambiae sensu stricto Malaria Mosquitoes in Response to Human Odor and Heat
Source: PLoS One. 2013 May 2;8(5):e62995. doi: 10.1371/journal.pone.0062995 (PMC3642193; doi:10.1371/journal.pone.0062995)
Supplement: Table S2 — ANOVA for main effects and interaction of treatments on the tangent (crosswind-behavior) for different distances to the upwind screen. (DOCX) [file pone.0062995.s006.docx]

**Table S2. ANOVA for main effects and interaction of treatments on the tangent (crosswind-behavior) for different distances to the upwind screen.** The mean (± s.e.m) tangent is given per section followed by main effect and interaction estimates for each treatment. Results are given for the *xy-* and *xz* plane respectively and indicated with * for different levels of significance. Signif. codes: < 0.001 '***' ,0.001 '**', 0.01 '*' .

| *xy* | Section I - IV  0-60 cm | Section I  0-15 cm | Section II  15-30 cm | Section III  30-45 cm | Section IV  45-60 cm |
| --- | --- | --- | --- | --- | --- |
| Mean (s.e.m.) tangent | 0.862 (0.024) | 1.051 (0.026) | 0.696 (0.037) | 0.625 (0.039) | 0.567 (0.037) |
| Odor main effect | 0.088*** | 0.057* | 0.118** | 0.116** | 0.133*** |
| Heat main effect | 0.061* | 0.073** | 0.006 | 0.074 | 0.057 |
| Odor.heat interaction | -0.003 | -0.027 | 0.050 | 0.038 | 0.034 |
| *xz* | Section I - IV | Section I | Section II | Section III | Section IV |
| Mean (s.e.m.) tangent | 1.311 (0.037) | 1.677 (0.041) | 0.903 (0.044) | 0.789 (0.050) | 0.777 (0.048) |
| Odor main effect | -0.011 | -0.105* | 0.113* | 0.104* | 0.125* |
| Heat main effect | 0.018 | 0.013 | 0.013 | 0.044 | 0.014 |
| Odor.heat interaction | -0.040 | -0.030 | -0.025 | 0.005 | 0.021 |
