# Supplementary material for: A 3D Analysis of Flight Behavior of Anopheles gambiae sensu stricto Malaria Mosquitoes in Response to Human Odor and Heat
Source: PLoS One. 2013 May 2;8(5):e62995. doi: 10.1371/journal.pone.0062995 (PMC3642193; doi:10.1371/journal.pone.0062995)
Supplement: Table S3 — The mean difference of ‘ x in’ - ‘x out’ presented for each mosquito while entering the plume and the mean difference of ‘x out’ - x in’ upon exiting. (DOCX) [file pone.0062995.s007.docx]

**Table S3. The mean difference of ‘*x* in’ - *‘x* out’ presented for each mosquito while entering the plume and the mean difference of *‘x* out’ - *x* in’ upon exiting.** A positive mean represents upwind progress. n Represents the number of occurrences within the recorded track.

|  | Entering  Difference ‘x in’- ‘x out’ | | | Exiting  Difference ‘x out’- ‘x in’ | | |
| --- | --- | --- | --- | --- | --- | --- |
| Mosquito  ID | n | Mean  (mm) | s.e.m. | n | Mean  (mm) | s.e.m. |
| 42 | 131 | 27.08 | 6.07 | 112 | -6.49 | 7.98 |
| 43 | 19 | 29.53 | 24.71 | 14 | 2.57 | 5.52 |
| 44 | 71 | 21.76 | 9.99 | 70 | -21.79 | 10.92 |
| 52 | 4 | 8.50 | 8.39 | 3 | 13.00 | 5.29 |
| 57 | 46 | 31.87 | 13.86 | 36 | -16.08 | 8.20 |
| 60 | 6 | 92.33 | 93.96 | 6 | -14.00 | 22.00 |
| 62 | 4 | 123.50 | 171.25 | 2 | 21.00 | 26.00 |
| 64 | 16 | 35.56 | 20.81 | 14 | -8.79 | 3.52 |
| 65 | 33 | 31.15 | 18.38 | 29 | -2.31 | 6.78 |
| 66 | 11 | 81.09 | 62.87 | 11 | -38.64 | 25.69 |
| 67 | 9 | 25.67 | 19.44 | 8 | 60.38 | 28.85 |
| 69 | 114 | 37.17 | 7.40 | 83 | 0.94 | 5.31 |
| 70 | 17 | 5.71 | 7.76 | 17 | 4.71 | 3.27 |
| 78 | 48 | 9.44 | 16.04 | 31 | -6.90 | 4.85 |
| 79 | 35 | 19.69 | 7.74 | 22 | 11.36 | 4.42 |
| 81 | 26 | -9.77 | 10.06 | 17 | -65.53 | 51.92 |
| Total | 590 | 27.36 | 3.75 | 475 | -8.11 | 3.49 |
