# Supplementary material for: A 3D Analysis of Flight Behavior of Anopheles gambiae sensu stricto Malaria Mosquitoes in Response to Human Odor and Heat
Source: PLoS One. 2013 May 2;8(5):e62995. doi: 10.1371/journal.pone.0062995 (PMC3642193; doi:10.1371/journal.pone.0062995)
Supplement: Text S1 — Air treatment system. (DOCX) [file pone.0062995.s008.docx]

**Text S1**

**Material and Methods**

*Air treatment system*

An air treatment system and wind tunnel was developed and installed by Facility Services Tupola, Plant Sciences Group, Wageningen University and Research Centre (WUR), The Netherlands. The system ensured a constant flow of purified warm and humid air into the wind tunnel (Fig.S1). Air collected outdoors was led through a series of filters driven by a fan based on ECM technology (P. Lemmens Company N.V., Gembloux, Belgium). After a primary dust filter, the air passed an Opakfil Green® fine dust filter (Camfil Farr, La Garenne-Colombes, France). Four activated charcoal filter cartridges were used for further purification of the air. Pressure differences over filters were measured to monitor the filter conditions. Purified air was pre-heated by a heating element (Bo-air, Drunen, The Netherlands) and led into a closed circuit existing of an 8 m long tube, 60 x 60 cm wide. Inside the closed circuit (the shunt), preheated air passed over an ultrasonic humidifier (Stulz Gmbh, Hamburg, Germany). As a result of adiabatic cooling, the desired temperature was reached and relative humidity increased towards saturation. At the end of the shunt the saturated air was mixed with the filtered outdoor air. Within the cooling or heating section (Bo-air, Drunen, The Netherlands) the air was tuned to achieve the desired conditions. The air output section consisted of a diaphragm valve to ensure a constant flow towards the wind tunnel and a manual air valve for air inlet to the duct with the pre-heating section. The air treatment system was computer-controlled using EXOcompact technology (RT2000 B.V., Waardenburg, The Netherlands. All experiments were conducted with air of 27± 1 ˚C and a relative humidity of 70 ± 3 %. The wind speed in the arena was 200 ± 10 mm/s.

*Wind tunnel*

The side walls and floor of the arena were constructed of black recycled polycarbonate and the ceiling was made of transparent Lexan polycarbonate (WSV Kunststoffen, Utrecht, The Netherlands) (Figure S2). A plastic container (diameter 5 cm, height 3 cm) holding a single female mosquito was placed at the downwind end of the flight arena, exposing the mosquito to the airstream when the lid was removed. The bottom of the container was made of meshed metal gauze to allow air to pass through. Flight images were recorded using the Noldus MPEG Recorder 1.0 software (Noldus Information Technology, Wageningen, The Netherlands) that digitized the images from the cameras using an encoder board installed on the PC.

Four Tracksys (Nottingham, UK) infrared light units were placed at the front of the wind tunnel, facing the air flow. Each unit contained an array of 90 infrared LEDs emitting light with peak output at a wavelength λ of 880 nm. To optimize lighting conditions, four IR lights containing 168 LEDs (λ > 920 nm) each (Reinaert Electronics, Amsterdam, The Netherlands) were added in the same line as the Tracksys IR lights. The reflection of IR light from the mosquitoes’ wings was filmed with two Cohu 4722-2000/0000 monochrome CCD video cameras (Cohu, San Diego, CA,USA) with Fuji non-tv f1.4 9 mm lenses, synchronized within 0.01 s. The cameras were mounted at 120 and 110 cm from the top of the wind tunnel, respectively, with their optical axes at an angle of 40 degrees. This solution minimized the reflections on the transparent top wall of the wind tunnel. To enable spatial orientation of the mosquito, low light conditions were provided by two incandescent lights (7 & 15 Watt) facing away from the arena. Light level in the human visual spectrum was less than 0.74 Lux measured at the center top of the arena (Apogee instruments Inc., Logan, UT, U.S.A.).

*Temperature measurements*

At completion of the experiments, the effect of the heat element on the air temperature on the upwind side of the screen was measured using a TC-08 thermocouple data logger (Pico Technology Ltd, St Neots, U.K.) connected to five type K thermocouples. During measurement series 1 and 2, the thermocouples were placed in a horizontal line with the heat source, starting at the upwind screen of the flight arena. To demonstrate the effect of convection, one series of readings was conducted in the positive z direction under an angle of

13 degrees, with intervals of 5 cm, measured from the top of the heat source. The angle of 13 degrees was chosen as a result of plume simulations using the Safex® fog generator. After calibration of the thermocouples, the mean of 60 readings (1 Hz) was calculated for each thermocouple (Figure S3).
