# Supplementary material for: A 3D Analysis of Flight Behavior of Anopheles gambiae sensu stricto Malaria Mosquitoes in Response to Human Odor and Heat
Source: PLoS One. 2013 May 2;8(5):e62995. doi: 10.1371/journal.pone.0062995 (PMC3642193; doi:10.1371/journal.pone.0062995)
Supplement: Text S2 — Computational methods to produce 3-D track data. (DOCX) [file pone.0062995.s009.docx]

**Text S2**

*Computational methods to produce 3-D track data*

For a precise 3-D reconstruction of a mosquito flight, Track3D required input for lens correction, calibration, filtering and interpolation. Track3D accommodated the air velocity and odor plume as created for the experiments; it produced 3-D target coordinates, accuracy checks for calibration and a 3-D reconstruction. Flight parameters (definitions in Table S1) could be calculated and presented per mosquito track in Microsoft Excel (Microsoft Office Professional edition, 2003) output files. The target path was reconstructed in a 3-D graph using different markers for positions inside or outside the plume (Figure 1, S3).

To reduce noise, the 2-D coordinates of tracked mosquitoes were smoothed by a Butterworth filter in combination with the zero phase shift routine filtfilt from Matlab 7.0 (Mathworks) with settings ‘filter order 2’ and cutoff frequency 8 Hz. Missing values, if not more than four in a row, were filled in by interpolated values. To this end, third order spline functions were used for all coordinates in a coherent block of data. Ideally, rays from camera centers to target projections on images should intersect at the 3-D target position. In reality there will be a distance between the rays, called the intersection error. The error was expressed in pixels, and only the smaller of the two values (one value for each camera, because of different scales) was used. 2-D coordinates which deviated more than 67 pixels from neighboring coordinates were deleted. This removes most artifacts such as reflections that were taken as target. The threshold of 67 pixels would theoretically result in a flight speed of > 200 cm/s relative to the walls with the assumption that there is no change in *z* direction.

Lens correction was applied by taking single pictures of a checkerboard in 20 different positions and using Matlab software from http://www.vision.caltech.edu/bouguetj/calib_doc/index.html to find for each camera the parameters PP (principal point), FL (focal length), and *k_c_* (coefficients of *r^2^* and *r^4^* for radial distortion where r is the distance from the center of the image). The relationship between normalized distorted coordinates *x_d_* and normalized corrected coordinates ***x_n_*** is ***x_d_****=****x_n_***(*1+k_c1_·r^2^+k_c2_·r^4^*), with *r^2^=****x_n_^2^*** (***x_d_****,* ***x_n_*** and ***x_p_*** are 2D vectors). Where the reverse relationship was needed, the corresponding parameters *k_ci1_* and *k_ci2_* were numerically approximated. *x_n_* follows from the pixel coordinates *x_p_* as:

***x_d_*** *=*(***x_p_****-*PP) /FL

*r^2^=****x_d_****^2^*

***x_n_****=****x_d_***(*1+k_ci1_·r^2^+k_ci2_·r^4^*).

After positioning of the cameras above the wind tunnel a calibration object of 60 x 58.5

x 57.5 cm was placed inside the flight arena. The object, made of black epoxy aluminum, had

28 white markers of circular shape (Ø = 15 mm) distributed in two levels and with known 3-D coordinates. Markers on each calibration image (for each camera view) were indicated in a fixed order, after which Track3D was used to determine the marker centroids. From the sets

of 2-D and known 3-D coordinates, DLT parameters were calculated as defined by

*u=* (*L1x+L2y+L3z+L4*)/(*L9x+L10y+L11z+1*)

*v=* (*L5x+L6y+L7z+L8*)/(*L9x+L10y+L11z+1*),

where *u* and *v* are the 2-D, and *x*,*y*,*z* are the 3-D coordinates. The 11 DLT parameters follow by application of standard linear algebra [1,and references therein]. Each camera had its own set of parameters.

The calibration results were checked in two ways. First, the known 3-D coordinates were combined with the DLT parameters to calculate the expected 2-D marker coordinates. These were compared with the measured camera coordinates. Second, the 3-D coordinates of the calibration markers were calculated from the measured 2-D marker coordinates and the DLT parameters. They were compared with the known 3-D coordinates. The calibration accuracy of the set-up was 0.5% of the dimensions of the tracking arena.

*Reference*

1. Groot, JH de, Leeuwen, JL van (2002) Estimation of the longitudinal axis of line symmetrical soft bodies by stereophotogrammetry. J of Biomech 35: 823 - 827. (doi:10.1016/S0021-9290(02)00017-9)
